# Supplementary material for: Glucocorticoid Regulates the Synthesis of Porcine Muscle Protein through m6A Modified Amino Acid Transporter SLC7A7
Source: Int J Mol Sci. 2022 Jan 10;23(2):661. doi: 10.3390/ijms23020661 (PMC8775876; doi:10.3390/ijms23020661)
Supplement: Supplementary file 1 [file ijms-23-00661-s001.zip › ijms-1519922-supplementary.pdf]

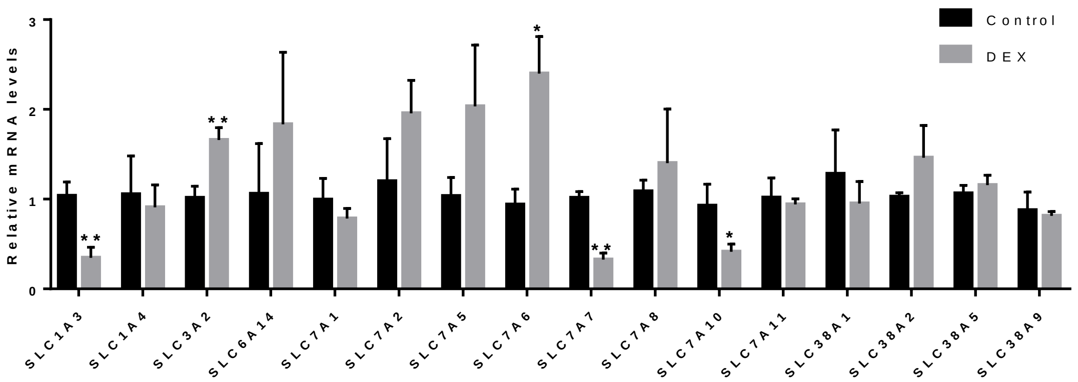


**Figure S1 mRNA expression levels of amino acid transporters**

Please find your results below. You may also download the results in plain text from here. Note that the results will be automatically deleted after 72 hours!

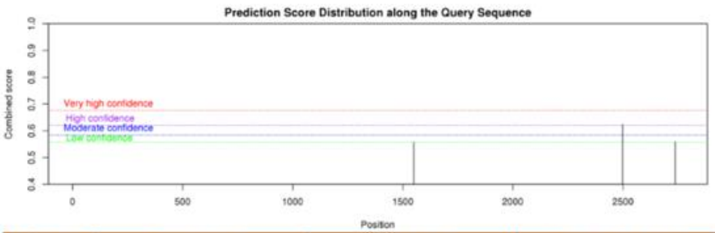


| # | Position | Sequence context | Structural context | Local structure visualization | Score(binary) | Score(knn) | Score(spectrum) | Score(combined) | Decision |
|---|---|---|---|---|---|---|---|---|---|
| 1 | 1550 | CUCUU CUCUU ACUCG<br>GGCUG GGACA CCCUC<br>AACUA UGUCA CUGAA | PHHHH HHHHH HHHHP<br>PPPPP PPMMM MMMMM<br>MMMMP PPPPP IPPIP | Draw | 0.535 | 0.521 | 0.597 | 0.559 | m6A site (Low confidence) |
| 2 | 2498 | AGGCA GCUUC ACCAA<br>CCUCU GAACU UGCCU<br>UUUGA GAAAU GGAGA | PPIIP PPPPM MMMMM<br>PPPPP IPPPP PPPPI<br>IIPPP PPIIP PPPPP | Draw | 0.671 | 0.597 | 0.560 | 0.623 | m6A site (High confidence) |
| 3 | 2737 | CAGUC UCCUU UAGAG<br>ACAUG AAACU GUCAC<br>AGGUA CUGGG CAAUA | MMPPP PPHHH HHPPP<br>PPMMP PPPPM MMMMP<br>PPPPP PPPPM MMMMM | Draw | 0.578 | 0.495 | 0.541 | 0.559 | m6A site (Low confidence) |

GTTCCCCTTCCCGCACCTGCCCCTCCTCCTGAGCGCTGGTAGCTCCTCCTCCTCGCACCTGCCCTAAGGTCCCAGGACAGAGGGCAATGCCAGGAAGCTGCTG
AAGGCTTTCTTCTCCTCCTCCGCC**ATG**GTTGACGGCATGAAGTATGAGGTGGCCTCCCAGCACGAGAGGGACGCCTCCCCTTTGGCCGAGGAGGCCGGCCCCC

**Initiation codon**

GGTCGGAGCAGGTAAAGCTGAAGAAGGAGATCTCACTGCTGAACGGCGTGTGCCTGATTGTGGGGAACATGATTGGCTCGGGCATCTTCGTCTCCCCCAAAG
GCGTGCTCATATACAGCGCCTCCTTCGGCCTCTCCCTGGTCATCTGGGCCGTCGGGGGCCTCTTCTCTGTCTTCGGGGCCCTTTGTTATGCGGAACTGGGCAC
CACCATTAAGAAGTCTGGGGCCAGCTATGCCTACATCCTCGAGGCCTTCGGGGGACTCCTTGCCTTCATCCGACTCTGGACCTCCCTCCTCATCATCGAGCCCA
CCAGTCAGGCGGTCATTGCCATCACCTTTGCCAACTACGTGGTGCAGCCCTTCTTTCCCAGCTGCTTCGCCCCCTACGCTGCCGACCGCCTGCTGGCTGCTGCC
TGCATCTCCTCGACTCACTTTGAGAATGCCTTTGAGGGTTCATCATTTTCAACAGGTGACATCGCTCTGGCACTCTACTCAGCTCTCTTCTCTTACTCGGGCTG
GGACACCCTCAACTATGTCACTGAAGAGATCAAGAATCCTGAGAGGAATCTGCCCCTCTCCATTGGCATTTCCATGCCCATTGTCACCATCATCTACATCTTAA
CCAACGTGGCCTATTACACTGTGCTAGACATGAGGGACATCCTGGCCAGTGATGCTGTTGCTGTGACTTTTGCAGACCAGATTTTTGGAATATTCAACTGGAC
AATTCCAGTGGCAGTTGCATTATCCTGCTTTGGTGGCCTCAATGCCTCCATTGTGGCTGCTTCTAGGCTTTTCTTTGTGGGCTCAAGAGAAGGCCATCTCCCTG
ATACCATCTGCATGATTCATGTTGAGCGGTTCACACCGGTACCTGCTCTGCTCTTCAATGGTCTCATGGCGCTGATCTACTTGTGCGTGGA**GGACA**TCTTCCAG

**$m^6A$ modification site**

CTCATCAACTACTACAGCTTCAGCTACTGGTTCTTCGTGGGGCTCTCTATCGTGGGTCAGCTCTATTTGCGCTGGAAGGAGCCTGACAGGCCTCGTCCTCTCA
AGCTCAGCATTTTCTTCCCCATTGTCTTCTGCCTCTGCACCATCTTCCTGGTGGCTGTTCCACTCTACAGTGATACCATCAACTCCCTTATCGGCATTGGCATTG
CCCTCTCAGGCTTGCCCTTTTATTTCCTCATCATCAGAGTGCCAGAACACAAACGACCTCTTTGTCTCCGCAGGATTGTGGCATCCATCACATGGTACCTTCAG
GTCCTGTGTATGTCAGTTGCTGCAGAGATGGATTTGGAAGATGGAGGAGAGATGCTCAAGCAACAAGATCCCAAGTCTAATTAAACATCACAAGGAATCTTGA
GATGTGGAAAGCAGGGGCCTCTTTTATCTCCTACCAGCCAGAGCCAAGAAGTTGAGAGGAAAGCTCACTTCATTGGAGGCAGCTACTAGAAGCCTCCTAGGC
AGCTTCACCAACCTCT**GAACT**TGCCTTTTGAGAAATGGAGAGTAATTTATTTGTTTTGCTACATATTGTTCCAGGTTTCTTTTAAAGGGATGATAAAGCAGACT

**$m^6A$ modification site**

GTGATGGGGAGCATGCCAGGTGTGGGCTTGGTTGTTCTGGCAGCATGCTGCCATGTATTAAGATAGTCACTCACCTCTGGGGTGCCTGCCCATTCCTTTCTGT
TGAAAGATCTAATAATGCTCCAAATTTCCAGTCTCCTTTAGAGACATG**AAACT**GTCACAGGTACTGGGCAATAAAAGGGTATGATC

**$m^6A$ modification site**

**Figure S2 Prediction of *SLC7A7* $m^6A$ modification sites.**
